# Geochemistry, Speciation, and Health Risks from Potentially Toxic Elements in Street Dust of Mbarara City, Uganda

**DOI:** 10.3390/jox16030083

**Published:** 2026-05-08

**Authors:** Hassan Omary Kumenya, Irene Nalumansi, Christopher Angiro, Ivan Kiganda, Timothy Omara, Emmanuel Ntambi

**Affiliations:** 1 Department of Chemistry, Faculty of Science, Mbarara University of Science and Technology, Mbarara P.O. Box 1410, Uganda; binomaryhosam@gmail.com (H.O.K.); inalumansi@must.ac.ug (I.N.); 2 Department of Environment Compliance, National Environment Management Authority (NEMA), Kampala P.O. Box 22255, Uganda; cangiro23@gmail.com; 3 Department of Chemistry, College of Natural Sciences, Makerere University, Kampala P.O. Box 7062, Uganda; ivan.kiganda@gmail.com

**Keywords:** mobility factor, particulate matter, vehicular traffic, urban dust

## Abstract

In equatorial Africa, rapid urbanization has increased city populations and particulate matter emissions. Street dust is a visual indicator that can be used to track urban pollution. In the present study, the total concentration and speciation of 10 potentially toxic elements (PTEs; As, Cd, Cu, Cr, Ni, Mn, Fe, Pb, Co, and Zn) in dust (n = 36) sampled from three streets of Mbarara City, Uganda, were determined using Energy Dispersive X-ray Fluorescence and Inductively Coupled Plasma-Optical Emission Spectrometry. The concentration of PTEs (0.27–36,401.50 mg/kg) geostatistically indicated moderate to extremely high enrichment of Cd, Cu, and Co in street dust. According to principal component and hierarchical cluster analyses, As, Pb, Cu, Zn, and Cd originated mainly from anthropogenic inputs, Fe and Mn came from geogenic sources, while Cr, Ni, and Co were from both natural and anthropogenic contributions. The mobility of the PTEs followed a general trend, Zn > Co > Cd > Ni > Cr, with Zn and Co being more environmentally mobile. Human health risk assessments indicated that discernible non-carcinogenic health risks may result from ingestion of dust by both children and adults. Children could also experience cancer health effects through the same exposure pathway.

## 1. Introduction

Urbanization and industrialization have long been utilized as proxies for development. They are historically linked and are indicators of economic growth, modernization, and social change [1]. However, increased industrialization and urbanization also lead to an increase in population and environmental pollution of urban centers and cities [2]. Among the various environmental matrices, street dust is a dynamic reservoir and (visual) indicator of urban pollution [3,4].

Street dust is a complex mixture of pollutants such as particulate matter, potentially toxic elements (PTEs), tyre and road wear particles, fly ash from asphalt, polycyclic aromatic hydrocarbons, and micro- and nanoplastics [5,6]. It is thus both a sink and a secondary source of PTEs. Due to its high surface reactivity, the total concentration of PTEs in street dust tends to be higher in cities with heavy industries or high vehicular traffic [7,8]. Elements such as arsenic (As), lead (Pb), cadmium (Cd), chromium (Cr), copper (Cu), zinc (Zn), and nickel (Ni) are toxic and bioaccumulative environmental pollutants [9,10]. Chronic exposure to PTEs is known to cause adverse human health effects, such as renal dysfunction, cancer, cardiovascular diseases, and neurological disorders [11]. In urban settings, inhalation of resuspended particles, ingestion through hand-to-mouth transfer, and dermal absorption are the key exposure routes to street dust with PTEs [12].

Several studies have investigated the total concentrations of PTEs in urban dusts globally [7,13,14,15,16,17]. However, chemical speciation (i.e., the distribution of PTEs among different binding forms or phases) provides a more accurate measure of their mobility, bioavailability, and potential toxicity by distinguishing between easily exchangeable and tightly bound metal fractions [18,19]. This is important in tropical cities where high temperatures, variable rainfall, and diverse urban activities may alter the partitioning of PTEs and enhance exposure risks [19].

Uganda, the focal area of the present study, is a developing Sub-Saharan African country that has undergone rapid urbanization and industrial growth over the past two decades [20]. This has outpaced her environmental management and pollution monitoring infrastructures [21,22]. Kampala, Jinja, and Mbarara cities are the fastest-growing commercial and industrial centers in Uganda [22]. Mbarara City is the country’s fastest-growing town outside the Kampala metropolitan area [23]. Consequently, it has experienced increased vehicular traffic, urban sprawl, establishment of small-scale industries, unregulated waste disposal, and infrastructure expansion [24].

In Mbarara City, previous studies reported particulate matter, PTEs, and carbon monoxide concentrations in ambient air, which were found to exceed acceptable limits [25,26]. However, the concentration of PTEs in dust from this city, the associated exposure, and health risks remain unknown. High concentrations of PTEs were recently quantified in dust from streets, roads, and bridges of the Elgon region, Uganda [12]. This area is polluted by high vehicular traffic and cement manufacturing. In continuity, the present study investigated the total concentration and speciation of 10 PTEs in street dust sampled from Mbarara City, Uganda. Geochemical indices, human health risk models, and multivariate statistical methods were further used to investigate the ecological and health risks as well as the origin of the quantified PTEs.

## 2. Methods

### 2.1. Streets Studied and Their Clusters

**Figure 1:**
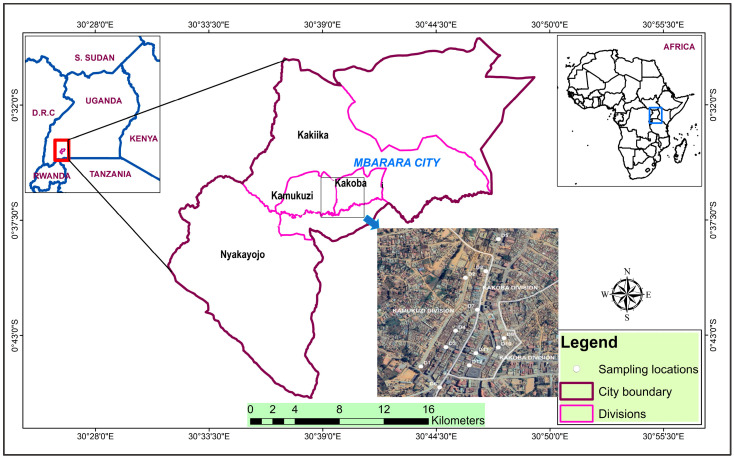
Map showing the sampled streets and their clusters in Mbarara City (detailed in Table S1). Inset is the location of Uganda in Africa (upper right). Map created using ArcGIS (version 11.5; Esri, Redlands, CA 909, USA).

The streets and their clusters considered were those in Mbarara, a rapidly growing, young city that attained its city status less than a decade ago (Figure 1). The city sits on 51.4 sq. km of hilly land rising up to 1430 m above sea level [26]. The city, with a recent estimated population of 221,300 people, is divided into the north and south constituencies [27]. These are further divided into six divisions, namely, Nyamitanga, Biharwe, Nyakayojo, Kakiika, Kakoba, and Kamukuzi [25].

### 2.2. Sampling and Sample Preparation

To preclude the influence of rain and wind, all the dusts were thus sampled in January 2024, following 1 week of continuous sunshine. To prevent exposure to PTEs in dust, safety gear was worn during sampling [12]. In total, 36 dust samples were obtained from Mackansigh, High, and Mbaguta Streets and their clusters using plastic brushes and dustpans (see Figure 1 and Table S1). In each case, 150 g of dust on the streetside or pavement were collected from areas of 0.5 to 1 m^2^. They were wrapped in aluminum foil and stored in labelled zip lock bags [12]. Samples were air-dried for 1 week in the laboratory and then dry-sieved through a stuck of 1000 and 63 µm meshes [7,8].

### 2.3. Physicochemical Characteristics of Samples

Electrical conductivity (EC) and pH of dust/water solutions (1:1, *w*/*v*) were determined using a combo waterproof pH/EC/TDS/temperature meter (HI 98129, Hanna instruments Inc., Woonsocket, RI 401, USA) [28]. Organic matter (OM) content was determined using a modified Walkley–Black titration procedure [29]. Briefly, sieved samples (1 g) were treated with 1 N potassium dichromate and concentrated sulphuric acid. The excess dichromate was back-titrated with 0.5 N ferrous sulfate heptahydrate solution, and a gradual colour change observed was from yellow to dark blue and finally to reddish purple at a sharp one drop end point. The average volume (*V_a_*) of ferrous solution was calculated from triplicate measurements and was recorded. Three blanks were run without dust samples to standardize the potassium dichromate, and the average volume (*V_b_*) was recorded. Total organic carbon (TOC) was calculated using Equation (1):

(1) $$\mathrm{TOC}\;=\;10\;[1\;-\;(\frac{V_{c}}{V_{b}})\;(N\;(0.003)\;(\frac{1}{w})]\times 100$$

where V_c_ = average volume of ferrous solution used to titrate the excess dichromate in the sample. The constant (0.003) is the milliequivalent weight of carbon (12/4000), used to convert the normality of the dichromate into grams of organic carbon; N = normality of potassium dichromate solution used for oxidation; 10 is the initial volume of the potassium dichromate in added to the sample; and W is the weight of dust sample analyzed.

To calculate the OM content of dust samples, Equation (2) was used, incorporating the Van Bemmelen factor of 1.724 [29]:

Organic matter (%) = TOC × 1.724(2)

### 2.4. Determination of Total PTE Concentrations

The total concentration of the PTEs (As, Cd, Cu, Cr, Ni, Mn, Fe, Pb, Co, and Zn) in 4 g of dust sample was analyzed non-destructively using an Energy Dispersive X-ray fluorescence (EDXRF) spectrometer (Nex CG II series, Applied RIGAKU Technologies Inc., Austin, TX 78613, USA), as detailed in its manual [30]. The covered plates were introduced directly into the EDXRF for elemental analysis. Spectrometer control and data analysis were achieved in QuantEZ^®^ software (Version 8). These total concentrations were compared with those obtained after digestion and followed by analysis on an inductively coupled plasma-optical emission spectrometer (ICP-OES; Optima 7000 DV, Perkin Elmer Inc., Waltham, MA 02451, USA), as described in a previous study [31].

### 2.5. Sequential Extraction and Assessment of Metal Mobility

The PTEs from the dust samples were fractionated in a five-step sequential extraction approach following a modified method from Tessier et al. [18], as described previously (Table S2) [19]. For fractionation, a 1 g portion was assigned after being weighed on an analytical balance. The extraction process comprised three steps, which were conducted in 50 mL polypropylene centrifuge tubes: leaching, separation, and washing. Centrifugation was used to separate the leachates and dust residual mixture for 30 min at 4000 rpm.

For ICP-OES analysis, the supernatant was pipetted into a 100 mL volumetric flask and diluted to the mark with double-distilled water. The mobility factor (MF), an index used to assess the potential mobility of metal ions in dust [19], was calculated using Equation (3):

(3) $$\mathrm{MF}\;=\;\frac{C_{F1}+C_{F2}+C_{F3}}{C_{F1}+C_{F2}+C_{F3}+C_{F4}+C_{F5}}\times 100$$

where C_F1_ to C_F5_ are the concentrations of the respective PTE in the five operationally defined fractions (F1 to F5 in Table S2).

### 2.6. Assessment of Street Dust Contamination and Toxicity Levels

Single-element contamination indices (enrichment factor, contamination factor, and index of geoaccumulation) were calculated using Equations (4)–(6) [8,32,33]:

(4) $$\mathrm{Enrichment}\;\mathrm{factor}\;(\mathrm{EF})\;=\;\frac{\left(\frac{C_{dust}}{C_{Fe}}\right)\;sample}{\left(\frac{C_{bkg}}{C_{Fe}}\right)\;crust}$$

(5) $$\mathrm{Contamination}\;\mathrm{factor}\;(\mathrm{CF})=\frac{C_{dust}}{C_{bkg}}$$

(6) $$\mathrm{Index}\;\mathrm{of}\;\mathrm{geoaccumulation}\;(I_{\mathrm{geo}})={log}_{2}(\frac{C_{dust}}{{1.5\;C}_{bkg}})$$

from which *C_dust_* = PTE total concentration; *C_Fe_ =* concentration of the reference element (Fe); $C_{bkg}\;$ = PTE background concentration (2, 0.102, 14.3, 35, 18.6, 527, 30,890, 17, 15, and 52 mg/kg for As, Cd, Cu, Cr, Ni, Mn, Fe, Pb, Co, and Zn) [34]; and 1.5 is the background matrix correction factor [35,36]. The dust pollution extents based on these indices are in Table S3.

The pollution load index (PLI) was calculated from the contamination factors (CF_As_ to CF_Zn_) using Equation (7) [37]:

PLI = (CF_As_ × CF_Cd_ × CF_Cu_ × CF_Cr_ × CF_Ni_ × CF_Mn_ × CF_Fe_ × CF_Pb_ × CF_Co_ × CF_Zn_)^1/10^ (7)

### 2.7. Assessment of Potential Health Risks

The risk model suggested by the US EPA was used [38] and determined for both adults and children. Consequently, the average daily doses (mg/kg/day) were estimated to establish exposure through direct ingestion (ADD_ingestion_), inhalation (ADD_inhalation_), and dermal contact (ADD_dermal contact_) with dust (Equations (8)–(10)) [39,40]:

(8) $${\mathrm{ADD}}_{\mathrm{ingestion}}=\frac{C\;\times \;IngR\;\times \;E_{f}\;\times \;E_{d}}{W_{ab}\;\times \;T_{aet}}\times 10^{-6}$$

(9) $${\mathrm{ADD}}_{\mathrm{inhalation}}=\frac{C\times InhR\times E_{f}\times E_{d}}{{PEF\times W}_{ab}\times T_{aet}}$$

(10) $${\mathrm{ADD}}_{\mathrm{dermal}\;\mathrm{contact}}=\frac{C\times S_{AF}\times CF\times AF\times {DAF\times E}_{f}\times E_{d}}{W_{ab}\times T_{aet}}$$

The exposure factors, their description, and values are detailed in Table S4. The hazard quotient (HQ) and hazard index (HI) were computed to determine the non-carcinogenic health risks posed by the PTEs (Equations (11) and (12)) [40]:

(11) $$\mathrm{HQ}\;=\;\frac{ADD}{R_{f}D}$$

(12) $$\mathrm{HI}=\sum_{i=1}^{n=10}HQ$$

The R*_f_*D is the oral (direct ingestion), inhalation, or dermal reference dose of the specific element.

The cancer risk (CR) was determined using the incremental lifetime cancer risk method for the carcinogenic PTEs (As, Ni, Pb, Cd, and Cr) (Equations (13)–(15)) [14,40,41]:

CR_ingestion_ = ADD_ingestion_ × CSF (13)

(14) $${\mathrm{CR}}_{\mathrm{inhalation}}={\mathrm{ADD}}_{\mathrm{inhaltion}}\;\times \;\frac{URF}{24}\times 10^{3}\times CSF$$

CR_dermal contact_ = ADD_dermal contact_ × CSF (15)

In the foregoing equations, URF is the chronic inhalation unit risk factor = 1.5 × 10^−4^, 4.9 × 10^−4^, 1.2 × 10^−2^, 4.3 × 10^−3^, and 1.2 × 10^−5^ per μgm^−3^ for As, Cd, Cr, Ni, and Pb, respectively [41,42,43,44]; CSF is the ingestion cancer slope factor = 1.5 × 10^0^, 5.0 × 10^−4^, 3.8 × 10^−4^, 3.0 × 10^−4^, and 8.5 × 10^−6^ mg/kg/day for As, Cd, Cr, Ni, and Pb, respectively [45].

### 2.8. Statistical Analysis

Analytical data were first subjected to Shapiro–Wilk test of normality. Further visual analysis was performed using Q-Q plots. They were averaged and expressed as mean ± standard deviation of quadruplicates, where the data were normal or median (interquartile range), where the assumptions of normality were not met. Significant differences in the mean values of physicochemical properties and concentrations of the PTEs in the dusts from the streets were established using one-way analysis of variance (ANOVA), followed by Tukey’s test or Kruskal–Wallis ANOVA followed by Dunn’s test for non-normal data. Spearman’s rank correlation analysis was performed to evaluate monotonic relationships between PTE concentrations and the physicochemical parameters of dust due to the non-normal distribution of the pooled data. Principal component analysis (PCA) was used to group the PTEs and apportion their origins, followed by hierarchical cluster analysis (HCA). All data visualization and statistical evaluations proceeded at *p* < 0.05 in Origin Pro 2026 (OriginLab Corporation, Northampton, MA 01060, USA).

## 3. Results and Discussion

### 3.1. Spatial Variations in Physicochemical Properties of Dust

**Figure 2:**
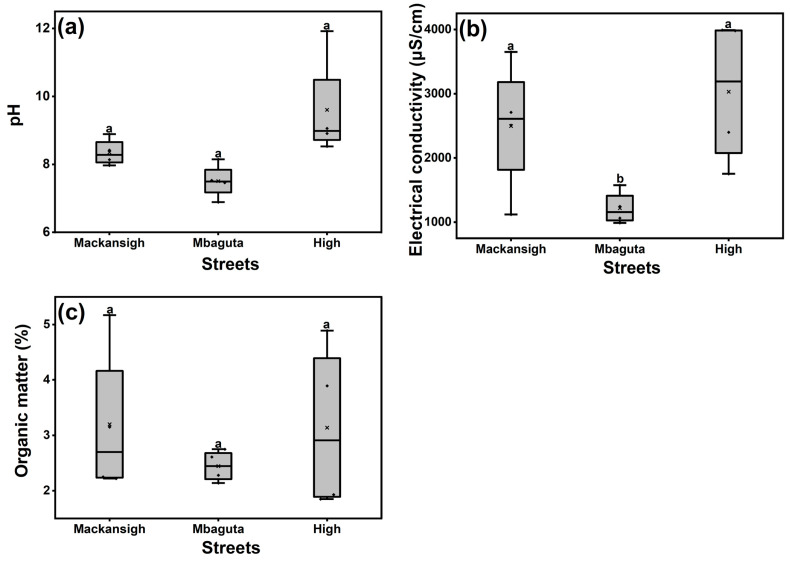
Mean values of the physicochemical properties of street dust in Mbarara City, Uganda (**a**) pH, (**b**) electrical conductivity, and (**c**) organic matter content. The box represents the interquartile range, and the line inside indicates the mean of four independent samples. Boxes carrying different alphabetical letters for a given parameter are statistically different as per one-way ANOVA (*p* < 0.05).

The analysis of selected physicochemical parameters of dust from the three streets recorded mean pH, electrical conductivity, and OM of 8.49 ± 1.26 (range: 6.89–11.92), 2249 ± 1138 μS/cm (range: 990–3990 μS/cm), and 2.93 ± 1.13% (range: 1.93–4.89%), respectively (Figure 2). The highest levels of the measured physicochemical parameters were in samples from Mbaguta Street, while the lowest were in samples from High Street. One-way ANOVA revealed that only the EC of dust samples varied significantly among the streets (*p* < 0.05).

The pH values ranged from slightly acidic to alkaline, with the highest pH value recorded in samples from Mbaguta Street. This is probably due to the fact that dust around this street is highly polluted by an influx of alkalizing substances such as detergents, particles formed by the abrasion of roadways, and those blown from construction sites and burnt agricultural wastes from Mbarara central market. In Moscow (Russia), the reported pH values of street dust were 6.4–8.1 [46] and 7.4–10.2 [47], which are comparable to results obtained in the present study. In Hyderabad (India), pH values of 8.01–10.66 were reported for street dust, which is well within the range found in the current study [48]. In the context of mobility, pH has a major effect on the dynamics of PTEs because it controls adsorption and precipitation, which are the main mechanisms of adsorption by most PTEs onto street dust [49]. For example, the mobility of PTEs within the dust profile decreases with increasing soil pH (8 and above) due to the precipitation of hydroxides and carbonates or the formation of insoluble organic complexes [50].

The highest EC value was recorded in samples from Mbaguta Street. This is due to the fact that the area is associated with agricultural wastes and heavy traffic. The measured EC values were very high, indicating that a considerable amount of the PTEs were present in the dust [51]. Previous studies reported EC values of street dust ranging from 112 μS/cm to 2500 μS/cm [48,52], which is lower than what is reported for dust from Mbarara streets.

The mean OM ranged from 1.85 to 5.17%, with the highest value quantified in samples from Mackansigh Street. These are comparable to the OM contents of 3.6–5.3% reported in street dust from Jordan and Liverpool [52,53]. It has been reported that organic matter content is important in the binding of PTEs to dust particles, and the solubility of Cd, Ni, and Zn is influenced by OM content of dust [54].

### 3.2. Spatial Variations of PTE Concentrations and Source Apportionment

The determined total concentrations of PTEs in street dust samples collected from the different streets of Mbarara City ranged from 0.27 mg/kg for Cd to 36,401.50 mg/kg for Fe. There were statistically insignificant differences (*p* > 0.05) in the concentrations of individual PTEs among the three streets (see Table S5). Of the PTEs, Cd, Pb, Co, and Zn occurred at concentrations 1.5- to 6.8-fold higher than their expected average concentrations in the continental crust, global shale, and upper continental crust [34,55,56]. Arsenic exceeded its average concentrations of 1.8 mg/kg and 2.0 mg/kg in the continental and upper continental crusts, while Cu, Cr, Ni, and Fe only exceeded their respective concentrations in the upper continental crust (Table 1). These concentrations were comparable to most reported PTE concentrations in street dust by previous studies in other cities worldwide. However, studies in cities such as Accra, Baghdad, Luanda, Mexico, and Tamale recorded very high concentrations of Cd, Cr, Ni, Pb, and Zn, with the concentration of Pb quantified at up to 1.8 orders of magnitude higher than in the present study [3,13,14,57,58].

**Figure 3:**
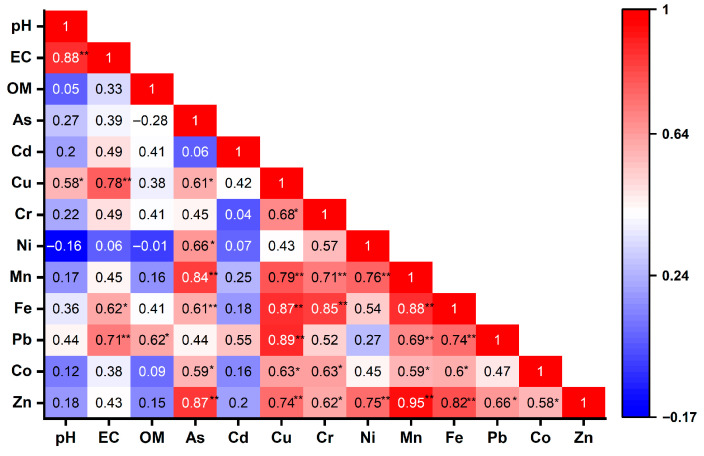
Spearman correlation coefficient matrix plot visualising the monotonic relationships between the physicochemical parameters and PTE concentrations of the dust samples. * Correlation is significant at the *p* < 0.05 level (2-tailed). ** is also significant at the *p* < 0.01 level (two-tailed). Colours indicate the strength and direction of Spearman’s correlation coefficient (ρ).

Source apportionment was guided by results from Spearman’s rank correlation analysis and multivariate statistical analyses (PCA and HCA). Significant monotonic associations between the PTEs and the physicochemical parameters of the dust samples were observed (Figure 3). For example, the EC showed strong positive monotonic relationships with Cu (ρ = 0.78, *p* = 0.003), Pb (ρ = 0.71, *p* = 0.01), and Fe (ρ = 0.62, *p* = 0.03), indicating that EC influences the PTEs contamination of Mbarara City dust. Furthermore, organic matter (OM) exhibited moderate monotonic associations with Pb (ρ = 0.62, *p* = 0.03), which could plausibly be due to its role in Pb binding and retention [62]. On the one hand, strong positive inter-metal associations were also observed among Cu, Pb, Zn, and As (ρ = 0.74–0.89, *p* < 0.05), indicating a common anthropogenic source. Similarly, Fe, Mn, and Cr exhibited strong associations (ρ = 0.85–0.88, *p* < 0.01), which could indicate geogenic origin.

On the other hand, Ni and Co showed moderate correlations with both groups, indicating that they originate from mixed sources. Weak negative monotonic associations were observed between Ni and both pH and OM, hinting that increasing pH and organic content promote the immobilization of Ni through adsorption onto mineral surfaces and complexation with organic matter [63]. Similarly, As exhibited a negative association with OM, which should rather indicate its preferential binding to mineral phases such as iron oxides rather than OM. These relationships indicate that physicochemical conditions influence the partitioning and mobility of the PTEs in the dust samples.

**Figure 4:**
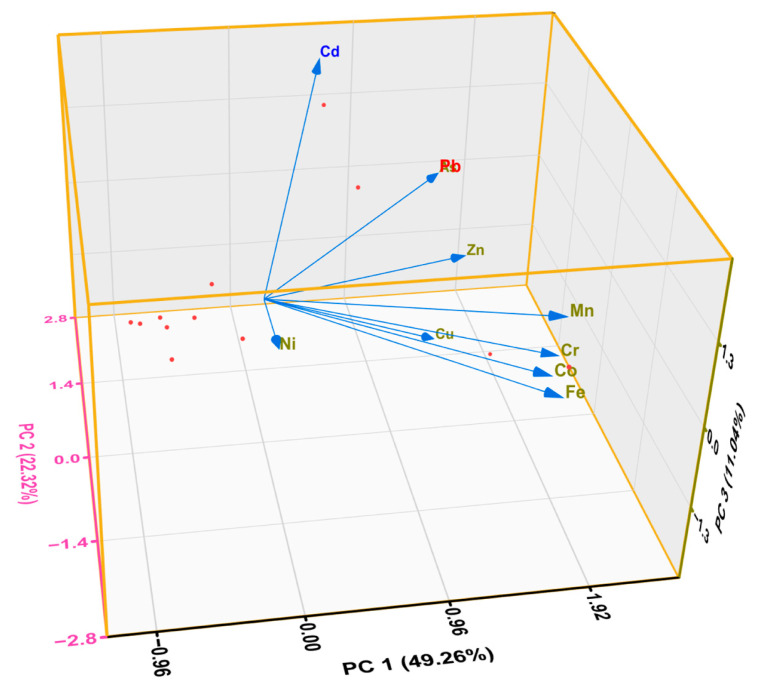
Loadings of principal components for the PTEs in street dust from Mbarara City, Uganda.

Furthermore, principal components from PCA explained up to 82.62% of the total observed variance (Table 2; Figure 4). The first principal component (PC1) accounted for 49.26% of the variance, with high loadings for Fe, Mn, Cr, Co, Zn, Pb, and As. This indicates a combination of geogenic contributions and anthropogenic inputs. The second component (PC2) explained 22.32% of the variance and was associated with As, Pb, and Cd, indicating the dominant influence of anthropogenic activities such as metal fabrication workshops and waste-related emissions. The third principal component (PC3), accounting for 11.04% of the observed variance, was characterized by a strong positive loading for Cd and a negative loading for Cu, indicating distinct source contributions.

**Figure 5:**
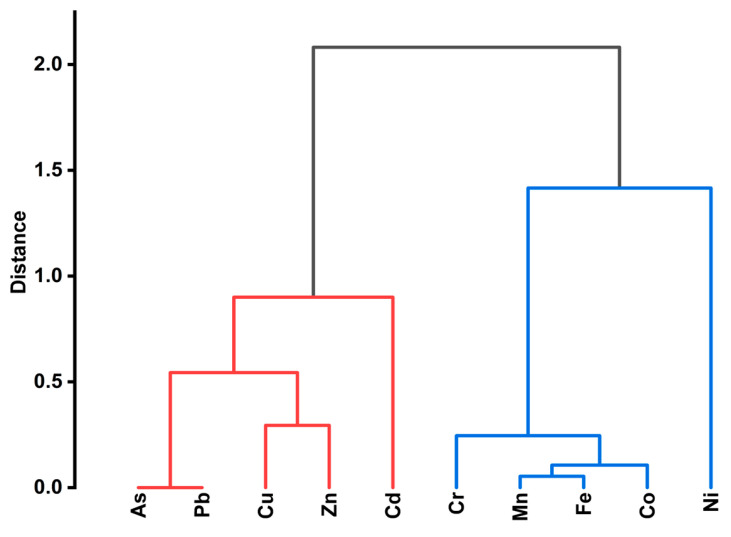
Dendrogram showing the clustering of PTEs in street dust from Mbarara city, Uganda.

The PCA results were supported by HCA, which revealed that the PTEs were grouped into two broad clusters (Figure 5). The first cluster was composed of Cu, Zn, Cd, Pb, and As, while the second cluster was constituted by Ni, Cr, Mn, Co, and Fe. The former cluster could be attributed to anthropogenic activities, such as disposal of lead-acid accumulators and batteries, paint from metal fabrication workshops, and urban emissions [31]. The second cluster consisted of PTEs mostly of geogenic origins, with Fe and Mn forming a tight subcluster. The association of Cr, Co, and Ni with the Fe–Mn subcluster suggests a mixed origin involving both natural and anthropogenic inputs or similarity in their environmental behaviors [41]. The clear separation between the two clusters implies that anthropogenic activities are the major contributors to PTEs’ contamination in the street dust.

### 3.3. Speciation and Mobility Factors of the PTEs

**Figure 6:**
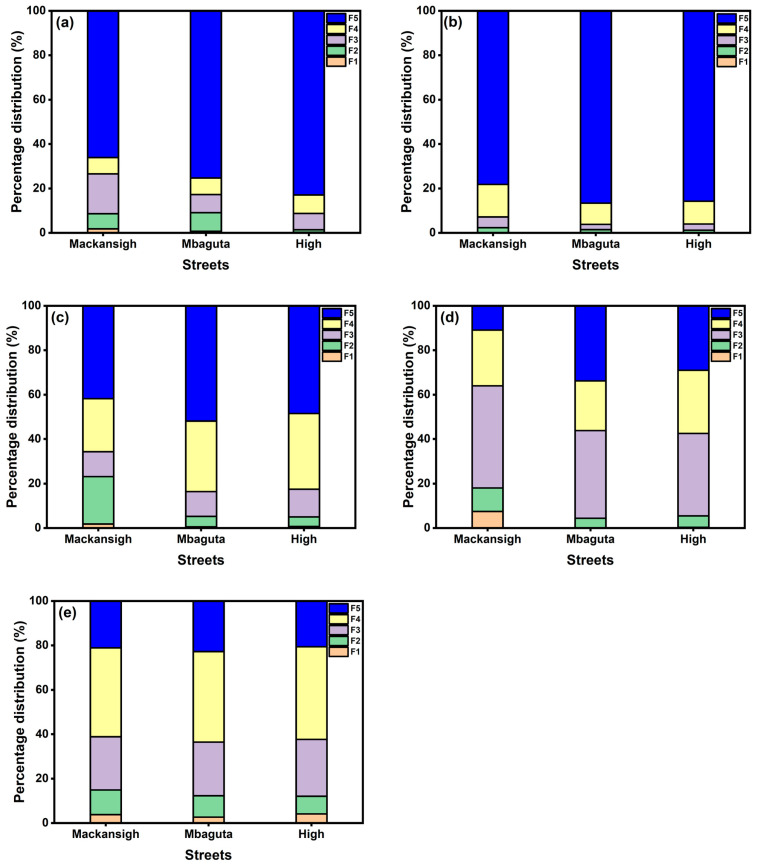
Percentage distribution of PTEs in the operationally defined fractions of dust samples (**a**) cadmium, (**b**) chromium, (**c**) nickel, (**d**) zinc, and (**e**) cobalt.

The sequential extraction results showed distinct distribution patterns of PTEs among the operationally defined fractions, with some variations among the studied streets. On the one hand, Cr and Cd were mostly associated with the residual fraction (F5; 77.9–86.4% and 66.2–83.1%, respectively), pointing to strong geogenic control and low mobility (Figure 6). Similarly, Ni tended to associate with the residual fraction (F5), and its presence in the organic-bound fraction (F4) should suggest it came from mixed sources, supporting the results of HCA [64].

On the other hand, Zn was dominant in the Fe–Mn oxide fraction (F3; 37.0–45.9%), implying that it can become mobile under changing redox conditions. Cobalt dominated the organic-bound fraction (F4; 40–42%) in all the samples, reflecting strong complexation with organic matter or humic substances [65]. Spatially, Mackansigh Street showed the highest proportions of PTEs in the more labile fractions, while High and Mbaguta Streets had PTEs in association with stable residual phases. Previous speciation studies reported that PTEs in street dust were mostly associated with the residual fraction (F5), which is considered the ultimate sink due to their attachment to the crystal lattices [19,66].

The streetwise order of PTEs’ mobility in the dust followed a general trend: Zn > Co > Cd > Ni > Cr, except for Mackansigh Street, where the portion of Cr was slightly greater than Ni (i.e., Zn > Co > Cd > Cr >Ni). Thus, Zn and Co are more environmentally mobile, while Cr is predominantly immobile due to its strong association with the residual fraction. It could be inferred from these results that while most of the PTEs in the dust samples were immobile, Zn and Co could pose a greater environmental risk due to their association with the more reactive fractions.

### 3.4. Environmental and Ecological Risk Assessment Results

**Figure 7:**
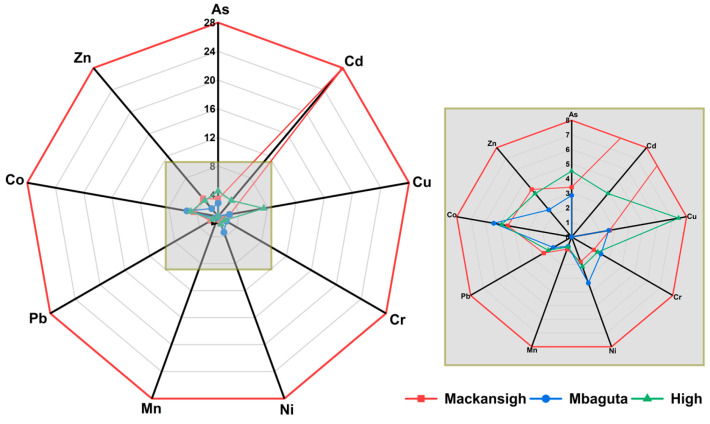
Radar plot showing the mean enrichment factors of PTEs in street dust of Mbarara City. Values were normalized from the mean of four independent samples using Fe as the reference element.

Among the PTEs, Cd was extremely enriched in dust samples from Mackansigh Street (EF = 28.1). Moderate to high enrichment was also observed for Cu (EF = 2.6–7.5), Co (EF = 4.5–5.4), and As (EF = 2.9–4.5) in the dust samples. Interestingly, Mn showed minimal enrichment (EF = 0.7–0.9), which should indicate that it is primarily of geogenic origin, as already shown by HCA results. The enrichment sequence of the PTEs was Cd > Cu > Co > As > Zn > Ni > Cr > Pb > Mn (Figure 7).

**Figure 8:**
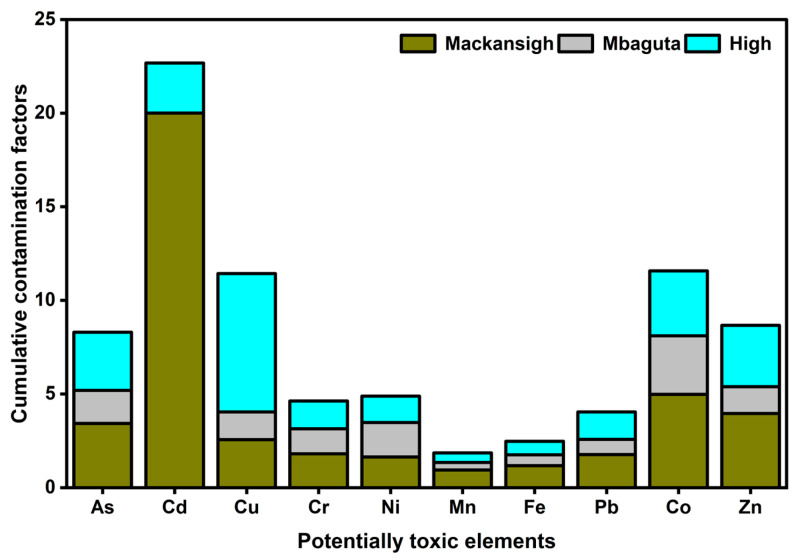
Contamination factors for the PTEs in street dust from Mbarara City. Values represent means of four independent samples.

The contamination factors ranged from 0.4 for Mn in samples from Mbaguta Street to 20 for Cd in the samples from Mackansigh Street (Figure 8). According to the classification advanced by Hakanson [33] (Table S3), there is low to very high contamination of dust from the studied streets in the order: Cd > Cu > Co > Zn > As > Ni > Cr > Pb > Fe > Mn. All the CFs fell within the range of 0.5–9.0 reported in previous street dust studies, except for Cd in dust from Mackansigh Street [12,19,67,68]. The PLI values calculated were 2.7, 1.2, and 2.2 for samples from Mackansigh, Mbaguta, and High Streets, respectively. By implication, all the dust samples could be categorized as polluted.

**Figure 9:**
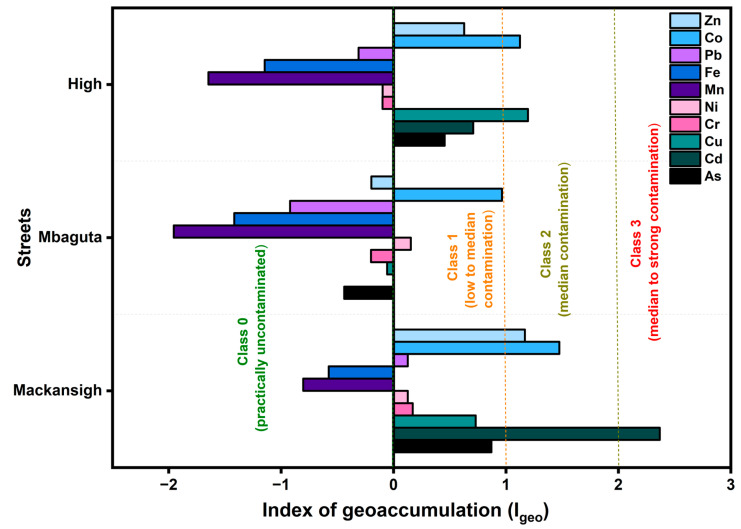
Index of geoaccumulation of PTEs in street dust of Mbarara City. Values represent means of four independent samples.

Based on the index of geoaccumulation values, spatial differences in contamination remained almost the same as already shown by enrichment and contamination factors (Figure 9). Mackansigh Street showed the highest contamination, with Cd showing median to strong contamination (class 3; I_geo_ = 2.37), while Co (I_geo_ = 1.47) and Zn (I_geo_ = 1.17) indicated median pollution (Class 2). Mbaguta Street was largely unpolluted (I_geo_ ≤ 0), with only slight enrichment observed for Co (I_geo_ = 0.96) and Ni (I_geo_ = 0.15). High Street showed median contamination for Cu (I_geo_ = 1.20) and Co (I_geo_ = 1.12), with minor contributions from Cd and Zn. In all the studied streets, Fe and Mn had negative I_geo_ values, which are often associated with PTEs of geogenic origins [12]. These results were in complete agreement with PCA and HCA results.

### 3.5. Human Health Risks Assessment Results

The average daily dose through ingestion of the PTEs in dust was from 4.55 × 10^−7^ mg/kg/day for Cd ingested by adults at High Street to 4.85 × 10^−1^ mg/kg/day for Fe ingested by children at Mackansigh Street (Table S6). The corresponding values for exposures through inhalation and dermal contact were 4.27 × 10^−11^ to 1.36 × 10^−5^ mg/kg/day and 5.45 × 10^−9^ to 2.18 × 10^−3^ mg/kg/day. The average daily doses did not surpass the reference doses for all the exposure routes. When inhalation and dermal contact exposure routes were considered, the calculated hazard quotients and indices never exceeded 1 for all the sampled streets and both age groups. However, the hazard quotients (4.14 × 10^−4^ to 6.93 × 10^1^) obtained for exposure through ingestion of PTEs in dust by both children and adults were higher than 1, with some values almost 70-fold higher. This implies that non-carcinogenic health risks could be associated with the ingestion of dust, with the highest probability in children. Two elements (Fe and Co) were identified as the primary drivers of this health risk, followed by As, Cr, Cd, Mn, Pb, Cu, Ni, and Zn. Consequently, all the hazard indices calculated for this exposure pathway were higher than 1 (4.50–72.97). These results are in agreement with previous studies in which ingestion pathway was the major route of exposure to PTEs in street dust [12,14,19,40].

The individual and total cancer risk values for both age groups (9.15 × 10^−17^ to 6.19 × 10^−7^) were within the US EPA safe range of 1 × 10^−6^ to 1 × 10^−4^ for exposure through dermal contact and inhalation (Table S7). Via ingestion, however, the street-specific and total cancer risk values obtained for children (7.10 × 10^−5^ to 1.38 × 10^−4^) were above the US EPA safe range. This health risk was largely contributed to by As, followed by Cr, Ni, Cd, and then Pb. Thus, children and other sensitive groups could presumably experience carcinogenic health effects if they regularly ingest dust from the studied streets of Mbarara City.

It should be noted that the health risk assessments presented were based on the total concentrations of the PTEs rather than those obtained from their sequentially extracted fractions. Cobalt was identified among the major contributors to the non-cancer risks, and according to speciation results in Section 3.4, it was distributed in relatively labile fractions. This makes it a more environmentally relevant PTE in Mbarara City.

### 3.6. Environmental and Public Health Implications

The quantification of PTEs in street dusts and the potential health risks calculated indicate the need for integrated strategies to reduce human exposure to street dust, which is a secondary emission source of resuspended PTEs inhaled by humans [4]. Effective control measures should thus combine (i) source reduction, especially traffic-related emissions and industrial inputs, with (ii) dust management options such as vacuum street sweeping and wet cleaning to remove fine particulate fractions [69]. Land use planning and control of construction dust emissions, as well as urban greening initiatives (e.g., parks, green roofs, urban forests, and living walls), can serve as natural sinks, filters, and bioindicators of particulate-bound PTEs [70]. The use of multiple strategies would ensure sustainable management of PTE concentrations in Mbarara City.

## 4. Conclusions

The present study assessed the contamination levels, distribution, and potential health risks of PTEs in dust from selected streets of Mbarara City, Uganda. Spatial variations were noted, with Mackansigh Street samples having the highest PTE concentrations, followed by those from High and Mbaguta Streets. Further assessment using the enrichment and geoaccumulation indices identified Cd, Zn, Cu, and Co as the most enriched PTEs, which were determined to originate from anthropogenic inputs based on multivariate statistical analyses. Ingestion of these PTEs could cause non-carcinogenic health effects in both children and adults, while carcinogenic health effects would be most probable in children. Together, the results showed that there is a combined influence of anthropogenic and geogenic sources on PTE concentrations in street dust of Mbarara City. It is therefore desirable to monitor and control urban dust pollution in rapidly growing and industrialized Ugandan cities. Future research should focus on size-fractionated and temporal dynamics of PTEs in the street dust and the evaluation of control measures, such as street cleaning and urban greening, to assess their effectiveness in reducing bioaccessible PTE fractions and the associated human health risks.

## Figures and Tables

**Table 1:** Concentration of PTEs (mg/kg) in dust from Mbarara City, as compared with previous studies in other cities.

City	As	Cd	Cu	Cr	Ni	Mn	Fe	Pb	Co	Zn	References
Mbarara, Uganda	3.53–6.86	0.27–2.04	22.65–35.50	46.60–63.37	25.85–30.40	210.25–499.61	17,813.0–36,401.5	13.73–30.14	46.85–74.76	79.55–150.01	This study
Mbale, Uganda	—	0.18–1.99	11.4–23.2	—	0.2–23.2	465–2630	—	185.0–244.0	—	26.8–199.0	[12]
Cairo, Egypt	0.81–8.65	0.16–1.38	12.56–351.4	15.53–67.97	1.06–36.28	140.97–746.58	24,571.8–16,377.9	54.2	—	184.2	[17]
Mexico City, Mexico	—	—	15–847.1	15–441	13.7–148.7	100–990.5	—	8.8–1907.8	—	38.7–4 827.6	[3]
Baghdad, Iraq	0.0006–0.0116	0.0004–0.0035	—	74.7–531.2	5.0–37.1	304.6–1829.0	—	1.4–50.3	10.7–61.5	55.7–1546.4	[58]
Al-Hillah, Iraq	11.8	0.2	57.3	301.0	75.5	482.0	25,600	64.6	12.1	168.0	[8]
Tamale, Ghana	—	24.9	56.1		11.5–18.5	502.4	2425	17.2	—	210.1	[57]
Accra, Ghana	—	—	29.0–76.5	123.8–220.7	6.5–15.9	235.9–379.6	19,782–36,630	33.6–117.5	—	124.5–371.7	[13]
Luanda, Angola	3.5–7.8	0.7–4.0	18.0–118.0	17–37	6.2–32.0	157.0–728.0	8000–20,100	74–1856	1.9–7.0	142–1412	[14]
Petra, Jordan	—	9.7	11.8	—	—	—	4694.4	31.6	—	24.8	[59]
Istanbul, Turkey	11.8	0.9	333.3	135.1	63.1	459.2	27,252	67.8	10.9	477.2	[7]
Abbottabad, Pakistan	—	0.24	50.0	13.0	10.3	304.0	15,540	21.5	6.66	139	[60]
Guwahati, India	—	0.37	18.07	22.76	108.73	229.25	11,559.4	7.94	4.37	68.31	[16]
Tiruchirappalli, India	—	—	11.84	11.47	—	—	506.39	0.24	—	47.08	[61]
Edinburgh, UK	—	3.3–4.1	81.5–107.6	—	—	—	—	112–268	—	64.2–101.4	[15]
Average continental crust	1.8	0.2	55.0	100	75.0	950	56,300	12.5	25	70	[55]
Global shale	13.0	0.3	45.0	90	68.0	850	47,200	20.0	19	95	[56]
Global upper continental crust	2.0	0.102	14.3	35	18.6	527	30,890	17.0	15	52	[34]

— means not determined.

**Table 2:** Total variance explained and principal component matrices for the PTEs in dust from Mbarara City.

Variable	Principal Components		
	PC1	PC2	PC3
As	0.3375	0.4043	0.0270
Cd	0.1314	0.3811	0.7019
Cu	0.3054	0.2081	−0.6280
Cr	0.3945	−0.2138	−0.0540
Ni	0.0024	−0.1591	−0.0041
Mn	0.3793	−0.2861	0.2613
Fe	0.3511	−0.3998	0.0651
Pb	0.3375	0.4043	0.0270
Co	0.3444	−0.3603	0.1041
Zn	0.3471	0.2050	−0.1590
Initial eigenvalues	4.93	2.23	1.10
Explained variance (%)	49.26	22.32	11.04
Cumulative variance (%)	49.26	71.58	82.62

## Data Availability

The original contributions presented in this study are included in the article/Supplementary Material. Further inquiries can be directed to the corresponding authors.

## Supplementary Materials

The following supporting information can be downloaded at: https://www.mdpi.com/article/10.3390/jox16030083/s1, Table S1: Description of the sampled streets and their clusters in Mbarara City; Table S2: Protocol for sequential extraction of trace elements from road dust; Table S3: Classification of the geostatistical indices used for the assessment of PTEs pollution of street dust from Mbarara City [17,32,33]; Table S4: Exposure factors used in health risk assessments of PTEs in street dust from Mbarara City [71,72,73,74,75]; Table S5: Summary of PTE concentrations in street dust and statistical analysis results; Table S6: Average daily doses, hazard quotients, and indices through ingestion, inhalation, and dermal contact with PTEs in dust from Mbarara City; Table S7: Carcinogenic risks due to ingestion, inhalation, and dermal contact with carcinogenic PTEs in street dust from Mbarara City.
